# The relationship between perceived social support and rumination among parents of children with autism: moderating effect of the degree of intervention received by children

**DOI:** 10.3389/fpsyt.2024.1340046

**Published:** 2024-05-07

**Authors:** Li Xu, Li Song, Zhiheng Xiong, Jiejia Chen

**Affiliations:** ^1^ College of Educational Science, Nanjing Normal University of Special Education, Nanjing, China; ^2^ School of Humanities, Southeast University, Nanjing, China; ^3^ School of Electronic and Information Engineering, Southwest University, Chongqing, China; ^4^ Key Laboratory of Cognition and Personality, Ministry of Education, Southwest University, Chongqing, China

**Keywords:** parents of children with autism, social support, rumination, intervention, mental health

## Abstract

**Objective:**

As the number of children diagnosed with autism rises year by year, the issue of nurturing this particular group becomes increasingly salient. Parents of autistic children, as the nearest and most reliable caregivers for their children, shoulder immense psychological strain and accountability. They are compelled to confront an array of daily life challenges presented by their children, as well as endure multiple pressures such as societal scrutiny and financial burdens. Consequently, the mental health status of the parents is of utmost significance.

**Methods:**

In this study, questionnaire survey combined with literature analysis were applied. The rumination thinking scale and the social support scale were used to investigate the relationship between social support perceived by parents of autistic children and rumination. Meanwhile, the moderating effects of intervention on children with autism were also explored. It hopes that our research would provide a basis for alleviating psychological stress and improving the mental health levels of the parents. A total of 303 parents of children with autism were collected (including 160 females and 143 males). Corresponding data analyses were conducted using SPSS 26.0.

**Results:**

Parents of autistic children generally exhibited high levels of rumination, with significant gender differences. At the same time, the perceived social support by the parents significantly influenced their level of rumination. It showed that the higher the social support received by parents, the lower the level of rumination. More importantly, the extent of intervention received by the children had a regulating effect on rumination of their parents.

**Conclusion:**

The personalized psychological support programs should be developed based on the actual situation of parents, to better manage the challenges presented by raising a child with autism. Our findings would provide important theoretical underpinnings and practical guidance for psychological intervention efforts aimed at families of autistic children. Moreover, these findings offer novel insights for future research, with the potential to advance the field of mental health studies concerning parents of children with autism.

## 1 Introduction

Autism spectrum disorder (ASD) encompasses a range of neurodevelopmental conditions characterized by social communication difficulties, language communication disorder narrow areas of interest or activity, and repetitive stereotyped behaviors (1). ASD not only affects the psychological health of children, but exerts a far-reaching impact on the psychological well-being of parents or guardians tasked with providing care. In terms of the responsibilities and duties in raising children, parents of autistic children should be understood as civilly competent adults who are related to the children by blood or legally recognized dependency. The parents have a duty to support the children, and are responsible for their behaviors (2).

Compared to caregivers of typically developing children, parents of autistic children tend to face more psychological difficulties, with a high risk of psychiatric disorders such as somatization, obsessive-compulsive states, repression, paranoia, and irritability (3). The sense of isolation and stress experienced by these parents are more profound than that of parents raising non-autistic children (4). Furthermore, parents of autistic children also exhibit elevated levels of caregiving stress than others (5). In a word, parents of autistic children are more likely to suffer from stress and mental health disorders (6). Nevertheless, the psychological strain of caring for autistic children can have detrimental effects on both parents and children, potentially diminishing the positive impact of interventions (7).

It has been found that rumination and negative affect are interactive, which can predict changes in anxiety levels after sadness has been induced (8). Some scholars have also discovered that parents of autistic children are likely to employ rumination to deal with negative events (9), which stands as a major influence on their anxiety levels and depression (10). Upon receiving a diagnosis, parents are often faced with the challenge of reconciling their expectations with the reality of their child’s condition, leading to difficulties in acceptance and a tendency towards rumination. This discrepancy and skepticism will prolong negative emotional states in individuals, which may not easily dissipate over time. Instead, they can exacerbate rumination through sustained introspection and anxiety, leading to a cycle of heightened psychological distress (11). To investigate the psychological experiences of parents with autistic children, identify the contributing factors, and provide tailored support and intervention, can not only alleviate the negative mindset of these parents, but also alleviate their psychological distress, fostering an environment conducive to well-being and stability (12). In addition, it has the potential to alter the ineffective parenting practices of certain individuals, encouraging greater involvement in the educational rehabilitation of autistic children and ultimately enhancing the efficacy of interventions (13).

The concept of rumination was first introduced in Nolen-Hoeksema’s research, which sought to explain the notable gender disparities observed in individuals with depression. Nolen-Hoeksema characterized rumination as a maladaptive cognitive response, wherein individuals engage in repetitive negative thinking about the symptoms, causes, and implications of their negative emotional state without actively seeking solutions (14). Rumination is a persistent psychological characteristic characterized by continual and adverse reflection on past pain and its associated circumstances, leading to a deterioration in psychological well-being and an inability to disengage from negative emotions. This may result in a diminished interest in external communication and a cessation of participation in positive social interactions (15). Through the process of rumination, parents of autistic children may develop negative countermeasures that constantly deepens self-blame and blame from others (16). The concept of rumination, as defined by Nolen-Hoeksema in 1987 within the context of the reaction mode theory of depression, refers to the persistent focus on one’s depressive symptoms and the associated causes and consequences (17).

Social support, conceptualized as the subjective perception of potential help from non-professional individuals within formal or informal contexts (18), has been identified as a key protective element that bolsters mental resilience. It has been shown to play a vital role in alleviating negative emotions and enhancing mental well-being (19). Research indicates a strong correlation between social support and both physical and mental health, with the ability to mitigate negative emotions (20). Seeking social support during periods of low rumination has been found to be particularly effective in alleviating their symptoms of depression and anxiety (21). Research indicates that rumination plays a significant role in influencing negative emotions (8), particularly among parents of children with autism who tend to respond to negative events through rumination (22). Parents of children with autism exhibit a heightened need for social support compared to parents of typically developing children. However, parents of children with autism face greater challenges in accessing both material and emotional social support resources compared to parents of typically developing children (23).

Early intervention for exceptional children is to provide early identification, early detection, early diagnosis, and comprehensive services such as medical care, health care, rehabilitation, education, social services, and child care guidance for preschoolers with developmental deficits or at risk of having developmental disability and for their families (24). According to field investigations and verification from the official websites of multiple institutions, it is acknowledged and widely applied across various organizations in Mainland China that Applied Behavior Analysis (ABA) therapy is the intervention measure commonly used for children with autism. Research has demonstrated that ABA-based therapeutic approaches are the only treatment methods supported by extensive empirical evidence, buttressed by hundreds of single-case experiments and an increasing number of between-group studies (25). The early interventions for children with autism referred to in this study are all based on ABA. Studies showed that a scientific early intervention can do much to correct autistic children’s restricted repetitive behaviors (26), improve their social interaction skills (27), and develop their potentials. Furthermore, it can be much beneficial to the family and society (28). Parental counseling interventions play a crucial role in supporting parental adaptation (29), with parental levels of psychological stress being influenced by their perceptions of the impact of interventions on their children (30), rumination is positively correlated with their mental pressure (31).

Overall, three hypotheses were proposed in this study: (1) Parents of autistic children have high levels of rumination; (2) Social support has a negative correlation with rumination of parents of autistic children; (3) The intensity of interventions that an autistic child receives modulates the correlation between social support and the ruminative thinking of their parents.

## 2 Methods

### 2.1 Subjects

The study collected data from parents of children diagnosed with autism through various online groups on the Baidu Tieba platform, known for its widespread communication and popularity among this demographic. They are fathers or mothers of autistic children diagnosed by medical professionals, hailing from various provinces and regions across China, with a broad overall data distribution. They voluntarily participated in the questionnaire survey via the internet link, WeChat code scanning, and other methods, when researchers published questionnaire links in different online groups. This data collection period last 42 days. After obtaining informed consent from the parents of children with ASD, a total of 335 questionnaires were distributed. Following the exclusion of invalid data due to excessively short completion times, patterned responses, and incorrect answers to trap questions, 303 valid datasets were obtained, resulting in an effective questionnaire retrieval rate of 90.45%. The study involved 143 fathers and 160 mothers, with a mean age of 37.22 years (SD = 3.29). All children with ASD included in this study were diagnosed with the condition at a hospital, comprising 177 boys and 126 girls with ASD, with an average age of 12.08 years (SD = 3.78). Further details are provided in **Table 1**. This study was approved by the Ethics Committee of Nanjing Normal University of Special Education.

**Table 1:** Basic information of children with ASD and their parents(N= 303).

Variables	Categories	Number	Percentage
Gender of parents	Male	143	46.30%
	Female	160	51.80%
Gender of children	Male	177	57.3%
	Female	126	40.80%
Whether the child is an only child	Yes	56	79.90%
	No	247	18.10%
Educational background of parents	High school and below	183	59.20%
	Junior college or above	120	38.80%
Annual household income	30000-60000 RMB	155	51.20%
	60000-90000 RMB	83	27.40%
	Over 90000 RMB	65	21.50%
Duration of intervention	Less than one year	94	31.00%
	One year and above	209	69.00%

### 2.2 Tools

The basic information section of the self-administered questionnaire includes the parents’ gender, age, and annual household income; the child’s gender, age, only child or not, and the duration of the intervention. In this investigation, the threshold criterion employed is the acceptance of early intervention by parents of autistic children for a minimum duration of one year, which aims to examine the influence of such interventions on the ruminative thinking patterns of parents. Meanwhile, other researchers also adopted the annual timeframe as the basic unit for assessing the rehabilitative outcomes experienced by children with autism (32–34). Additionally, in a posttest study examining early intervention in children, it was found that by comparing the changes in the children before and after receiving the intervention for one year, the one-year duration allowed for a more accurate assessment of the intervention’s effects (35). Given the series of previous studies and the fundamental attributes of interventions for children with autism, such as significant individual differences, non-evident initial intervention effects, slow progression rates, and extended rehabilitation cycles (36, 37). Our study proposes the stratification of intervention intensity received by children with autism over one year. The rationale behind this stratification approach is rooted in the recognition that intervention treatment for children with autism is typically a protracted and intricate process, where significant effects may not be readily observable in the short term. By delineating interventions using a one-year boundary period, we can more accurately elucidate the characteristics and patterns of intervention treatment. This not only aids in understanding and describing the intervention process but also offers a valuable framework for subsequent research endeavors.

All questionnaire responses were provided by the parents.

Ruminative Responses Scale was initially created by Nolen-Hoeksema and her group. The present study used the version that has been tested for its reliability and validity, and generalized and revised by Han Xiu and other people in 2009 (38). The scale consists of 22 items dividing into three factors: symptom rumination, brooding, and reflective pondering. The symptom rumination dimension primarily concentrates on the individual’s recurrent contemplation of negative emotions and experiences. The worry dimension is oriented towards evaluating the individual’s apprehension regarding potential adverse events in the future. The reflective thinking dimension underscores the individual’s profound examination and comprehension of past occurrences. On a scale of 1-4 (1=never; 2=sometimes; 3=often; 4=always), the higher the score, the more serious the tendency of rumination. In this study, the Cronbach’sα coefficient of the scale was 0.98, and the internal consistency reliability of the three sub-dimensions was between 0.94 and 0.97.

The Social Support Rating Scale (SSRS), created by Xiao Shuiyuan in 1986 and chosen for this study, is relatively widely used in China (39). The scale consists of 10 items dividing into three basic aspects: objective support, subjective support and utilization of support. Objective support encompasses the tangible, material aid and practical assistance that an individual genuinely receives from their social network. Subjective support zeroes in on the emotional underpinnings of an individual’s perceived social connectedness, emphasizing the sentiments of care, understanding, and respect they experience. The capacity to utilize support effectively mirrors an individual’s ability to adeptly draw upon and harness the social resources at their disposal during times of adversity. These three dimensions offer an all-encompassing reflection of the stratified experiences individuals have within their social support networks. They capture not only the tangible aspects of received aid but also the deeper emotional currents of perceived care, understanding, and respect. For item 1-5 and 8-10, scores of 1, 2, 3 or 4 will be allotted respectively for choosing 1, 2, 3 or 4. For item 6 and 7, choosing “no source” will be scored 0, and choosing “the following sources” will be scored. More sources, higher scores. Higher scores on the scale indicate greater perceived social support from parents of children with autism. The study found a Cronbach’s α coefficient of 0.92 for the scale, with internal consistency reliability values ranging from 0.77 to 0.91 for the three sub-dimensions.

### 2.3 Data analysis

Data analysis was conducted using the SPSS 26.0 software package and the PROCESS macro program 3.3 developed by Hayes. According to the hypothesis of this study, the moderating effect was tested using model 1 in PROCESS (40). The questionnaire survey method was used to analyze the data of the study. To validate the three hypotheses mentioned above, questionnaire survey method together with literature analysis were employed to test and discuss them step by step.

## 3 Results

### 3.1 Preliminary analysis

Pearson product moment correlation was used to conduct correlation analysis of the main variables in this study, and the results are shown in **Table 2**. As it can be seen from **Table 2**, parents’ Perceived social support is significantly correlated with their children’s intervention degree positively, and significantly correlated with rumination negatively. Furthermore, gender and age exhibited significant correlations with children’s intervention degree, perceived social support, and rumination, thus warranting their inclusion as control variables in subsequent model analyses. As a result, the hypotheses 1 and 2 were confirmed.

**Table 2:** Descriptive statistics and correlations among variables.

Variable	*M*	*SD*	1	2	3	4	5
Gender	0.47	0.50	1				
Age	37.22	3.29	-0.17^**^	1			
SS	31.45	9.04	0.24^**^	-0.46^**^	1		
Rumination	42.55	19.19	-0.24^**^	0.49^**^	-0.96^**^	1	
ID	0.69	0.46	0.19^**^	-0.38^**^	0.68^**^	-0.70^**^	1

SS, Social Support; ID, Intervention Degree. Gender was dummy coded as female = 0, male = 1. * P <.05.** p <.01. *** p <.001.

### 3.2 Test of the moderation effect of social support and intervention

To investigate the moderating impact of children’s level of intervention on the association between social support and parental rumination in families of children with autism, Model 1 of the SPSS macro program PROCESS developed by Hayes was employed to examine the moderating effect of children’s intervention level (40). Firstly, gender and age were incorporated into the initial analysis to examine their impact on rumination. Subsequently, parental social support, child intervention level, and their respective interaction terms were included in a subsequent analysis to explore the predictive role of the moderating variable of child intervention level on rumination. The results are shown in **Table 3**.

**Table 3:** Testing the moderation effect of Social support and Intervention.

Predictor	Model (Rumination)			
	*β*	*t*	*β*	*t*
Gender	-0.17	-3.32^**^	-0.01	-0.47
Age	0.46	9.24^***^	0.05	2.61^*^
SS			-0.92	-37.35^***^
ID			-0.09	-4.54^***^
SS×ID			0.07	3.62^***^
*R^2^*	0.27		0.93	
*F*	54.85^***^		830.83^***^	

*P<.05. ** p <.01. *** p <.001.

**Figure 1:**
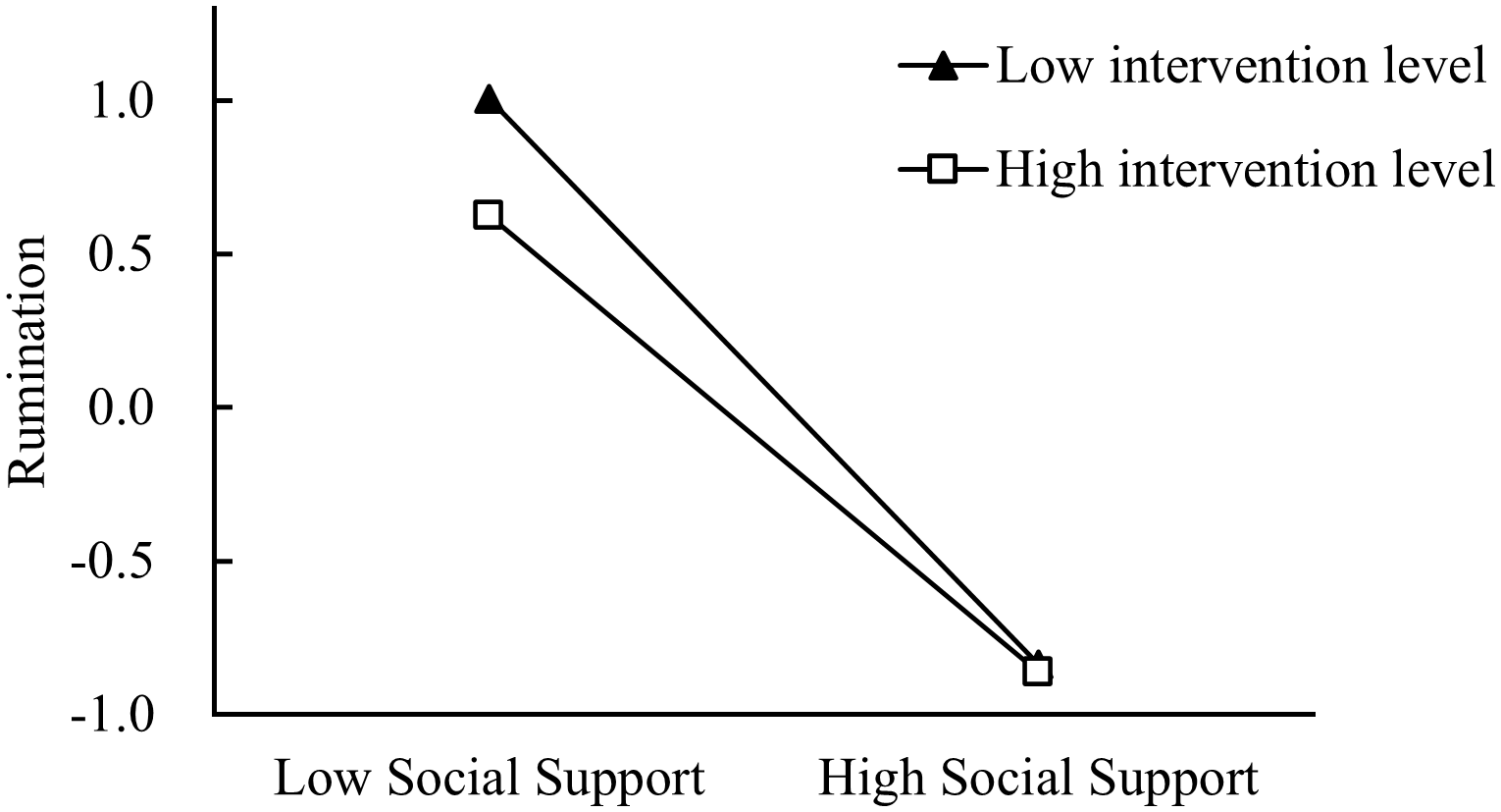
Interaction effect of Social support and Degree of intervention on the Rumination.

To further investigate the moderating impact of the level of child intervention on the association between social support and rumination as perceived by parents of children with autism, participants were categorized into two groups based on their level of child intervention: a high degree of child intervention group and a low degree of child intervention group. The predictive influence of social support perceived by parents of children with autism on rumination was then examined within each group. As shown in **Figure 1**, the results showed that the interaction term between social support and intervention degree significantly predicted rumination. As the simple slope tests shown, social support could significantly predict rumination at lower intervention degrees, *β* = -0.92, *p* < 0. 001; At higher intervention degrees, social support significantly predicted rumination, *β* = -0.74, *p* < 0. 001, but it was less forceful compared with the former predictions. As a result, the hypotheses 3 was confirmed.

## 4 Discussion

Parents of children with autism frequently encounter an array of parenting (41) stressors. The pressures faced by parents of children with autism encompass both subjective distress such as social stigma and feelings of helplessness, as well as objective challenges including a lack of medical and educational services and the financial burden of high rehabilitation costs. These pressures not only adversely affect the mental health of the parents but may also indirectly impact the physical and psychological adjustment and development of the children with autism. Understanding the psychological characteristics of parents raising a child with autism is particularly vital. In this study, we examined the interplay between parental perceived social support, rumination, and the level of intervention received by children with autism. We also delved into how the degree of intervention for the child with autism moderates these relationships. The outcomes of this research not only enrich our understanding of the intricate challenges faced by families managing ASD but also furnish valuable insights that can guide the formulation of more targeted and efficacious intervention and rehabilitation strategies in the future. By highlighting the importance of social support and the impact of intervention levels on parental well-being, this study underscores the need for comprehensive family-centered approaches to care for children with autism and their parents.

Through descriptive statistical analysis of rumination among parents of children with autism, it was observed that these parents generally exhibited elevated levels of rumination. Specifically, they are inclined to engage in rumination, worry, and self-blame in response to their child’s autistic symptoms, the treatment journey, and the everyday challenges they face, which may influence their emotional state and coping mechanisms. Furthermore, our investigation revealed a notable gender disparity in the propensity for rumination, with female parents demonstrating a greater proclivity towards rumination compared to their male counterparts. Consequently, hypothesis 1 of this study is confirmed. Research demonstrated that parents of children with learning disabilities showed higher levels of rumination in negative social situations (42). It has been documented that the parents are more likely to use rumination to cope with negative events (9). Nolen Nolen-Hoeksema argued that there were gender differences in rumination, with females showing more rumination than males (14). Research on rumination also revealed that men got lower scores on attachment anxiety, depressive symptoms, and obsessive-compulsive thinking than women (43). This suggests it is a shared phenomenon, regardless of cultural backgrounds. High levels of rumination in parents of autistic children may have much to do with the pressure of raising children (44), depression, and anxiety (45). With children with serious illnesses, their mothers, compared with their fathers, are more likely to discover changes happening to their children, and to engage in self-reflection (46). Due to heightened susceptibility to social expectations and gender roles, male parents may inadvertently conceal their profound negative emotions when responding to questionnaires, thereby masking their intrinsic vulnerability and sensitivity (47). This observed pattern of female superiority and male inferiority is attributed to variations in the social division of labor (48), long-term social education concepts, and societal expectations regarding emotional and personality traits between genders (49, 50).

In the correlation analysis between social support and rumination, it was discovered that all three dimensions of the social support scale—objective support, subjective support, and the degree to which parents utilize available support—were inversely correlated with the rumination experienced by parents of children with autism. This result indicates that there is a direct relationship between the amount of social support received by parents and their level of rumination: increased social support corresponds to decreased levels of rumination among these parents. Notably, among the three factors, “parental utilization of social support” exhibited the strongest correlation and had the most significant impact on rumination. These findings not only enhance our comprehension of the connection between social support and the psychological state of parents with autistic children but also offer a crucial theoretical foundation for subsequent psychological interventions and support strategies. Considering that “ parental utilization of social support” has the greatest influence on rumination, it can be inferred that enhancing parents’ capacity to recognize and utilize social support may be an efficacious method to diminish their rumination levels and ameliorate their mental health. Hence, hypothesis 2 of this study is corroborated. A negative correlation exists between the level of social support and rumination among parents of children with autism. The presence of effective and positive social support is shown to have a significant impact on individuals’ rumination tendencies, ultimately leading to positive outcomes (51). Recent studies also found that social support, rumination, and negative emotional states were negatively correlated, and that providing adequate social support is essential (52). According to some research, effective social support can make parents more capable to raise their children, help them to adapt new roles more quickly, and reduces their negative emotions such as anxiety and depression (53). Additionally, anxiety and depression symptoms exhibit a moderate association with rumination and also exert significant independent influences on excessive rumination (54). Furthermore, a noteworthy interaction between rumination and perceived social support is observed in daily life (55). Caregivers of autistic children may avoid social intercourse due to their strong sense of “stigma”. And they try to cover up this negative feeling by reducing or refusing social activities (56). Therefore, parents of autistic children will receive less social support, which will affect the level of rumination. Provide increased social support to mitigate rumination in parents of children with autism. Facilitating access to community-based emotional support and respect will effectively diminish feelings of anxiety and depression, alter negative thought patterns, and bolster parental confidence and problem-solving skills (57). It further has a positive impact on the rumination of parents of autistic children. In order to attain the objective, it is imperative to enhance awareness and education surrounding autism, foster greater societal acceptance of individuals with autism, and elevate the social standing of the autism community. It is crucial to consolidate all existing resources to establish a comprehensive social support system for children with autism, enabling their parents to enhance their communication skills and effectively utilize available social support services.

According to the results of the moderation effect analysis in this study, the impact of parental perceived social support on rumination is influenced by the degree of intervention received by children diagnosed with autism. This finding offers an understanding of the correlation between the degree of intervention for children with autism and the psychological state of their parents. Additionally, we examined the differences in rumination among parents of children who received varying degrees of intervention. Parents of children with autism who underwent higher degrees of intervention exhibited lower levels of rumination compared to parents of children with autism who received less intervention. Consequently, hypothesis 3 of this study is confirmed. The condition of autistic children and the effectiveness of interventions profoundly affect their parents’ rumination. For these parents, the core deficits of autism and the corresponding rehabilitation and educational programs are of their primary concern in the early stages of their child’s diagnosis. They want to know what to expect from their child’s future and how their child can get help and support (58). According to Goin-Kochel, the earlier autistic children were diagnosed, the more satisfactory their parents may feel. When children receive a diagnosis of autism at a later age, parents may exhibit increased negativity towards the diagnosis, intervention process, and subsequent rehabilitation outcomes (59). This is likely due to the inherent characteristics of autism, which result in minimal observable progress in the early stages of treatment, slow developmental advancements, and an extended rehabilitation timeline, ultimately diminishing parental confidence (60). Recent studies have demonstrated a significant correlation between rumination and negative emotions, particularly among parents of autistic children (8). This group is more susceptible to engaging in negative rumination when experiencing negative emotions (22), leading to increased self-blame and accusations. These patterns of rumination contribute to the development of personality traits characterized by negative emotions (16). Additionally, parents who engage in rumination exhibit lower levels of problem-solving confidence (61). Consequently, the rumination of parents of autistic children is significantly influenced by their children’s current behavioral challenges and future developmental expectations. It is necessary to improve parents’ scientific understanding of autism through professional training and daily popularization, so that they can discover the initial abnormal features of their autistic children as early as possible, take their children to the doctor in time and learn relevant knowledge. And after diagnosis, they should correctly recognize their children’s special behaviors so as to be able to better and faster find suitable intervention methods, and take better care of their children. Only when they see progress and hope in their children can the intervention have a positive regulation of rumination in parents.

However, our study has some limitations. First, the initial questionnaire utilized in this study primarily gathered data on fundamental demographics, such as gender and age, thereby hindering the ability to incorporate a nuanced analysis of cultural background distinctions. Subsequent investigations should prioritize the exploration of social and cultural variances to elucidate the factors contributing to varying levels of rumination observed among fathers and mothers from diverse cultural backgrounds. Second, the uneven distribution of parent samples in this study can be attributed to sampling difficulties. For future research, it is advisable to increase the size and number of samples while controlling for gender differences in order to increase the generalizability of the research findings. Third, it is important to note that the responsibility of raising an autistic child extends beyond just the father and mother, involving the entire family and various family systems. Therefore, it is recommended that future research endeavors include data collection from other caregivers, such as grandparents, to enhance comprehension of the correlation between social support and rumination. Fourth, with a focus on the facilitation of data analysis and the accentuation of research outcomes, this study divided the important influencing factors by year, which may overlook the precision of research data and the systematic nature of research results. therefore, subsequent studies by our team could be based on the theoretical foundations of early intervention for children with autism, allowing for a more refined and systematic analysis and research approach, such as dividing the duration of early intervention received by children with autism into three-month cycles. Such an approach would deepen our understanding of the effects of early intervention on children with autism and provide more scientific and specific guidance for practice in related fields.

## 5 Conclusions

In this study, a moderating model was introduced to deepen the understanding of the interaction between rumination and social support, which could contribute to the existing literatures. Firstly, it was found that social support significantly impacted the level of rumination among parents of children with autism. Specifically, the higher the level of social support, the lower the level of rumination among these parents. This finding not only helps us to understand the role of social support in alleviating parental psychological stress, but also provides an important basis for future psychological interventions for families with autistic children. Secondly, the study further revealed the moderating effect of the extent of intervention received by autistic children on the relationship between social support and parental rumination. It offers a novel insight that the degree of intervention may be a key factor influencing the effectiveness of social support, which would help us develop more accurate psychological intervention strategies to better meet the needs of families with autistic children.

## Data availability statement

The original contributions presented in the study are included in the article/supplementary material. Further inquiries can be directed to the corresponding author.

## Ethics statement

The studies involving humans were approved by The Ethics Committee of Nanjing Normal University of Special Education (application number: 20220518002). The studies were conducted in accordance with the local legislation and institutional requirements. The participants provided their written informed consent to participate in this study.

## Author contributions

LX: Writing – original draft, Writing – review & editing. LS: Writing – review & editing. ZX: Data curation, Writing – original draft, Writing – review & editing. JC: Writing – review & editing.
